# Disability and Symptom Burden Among the Very Old: Comparison of Survivors and Decedents

**DOI:** 10.1093/geroni/igaa057.744

**Published:** 2020-12-16

**Authors:** Xinran Liu, Steven Albert

**Affiliations:** University of Pittsburgh, Pittsburgh, Pennsylvania, United States

## Abstract

How does disability and symptom burden among the very old differ between those who die and those who do not die over 12 months? We explored patterns of disability and symptom burden in the Health ABC cohort study, which involved quarterly phone interviews in 2011-14 (years 15-17). A proxy completed the interview when the proband was unable to participate. We identified a sample of 291 decedents with at least 1-year of follow-up before death and matched a 1:1 sample of survivors at the time of death by race, sex, and age (within ±3 years). 252 decedents (age 90.0±3.03, 65.1% Black, 52.4% female) and 288 survivors (age 90.1±3.03, 64.9% Black, 52.4% female) with at least 3 quarterly interviews were included for analysis. Decedents had a higher proportion of proxy-reported interviews compared to survivors (40.9%vs16.0%, P<0.01). Disability prevalence among decedents was significantly higher (P<0.01) compared to survivors (using an assisted walking device, 62.3%vs37.4%; difficulty getting in/out of bed, 32.0%vs19.4%; difficulty bath/shower, 28.9%vs10.0%; difficulty dressing, 19.0%vs8.7%). Decedents and survivors differed significantly (P<0.05) in self-reported number of symptoms (2.35vs1.78), severity of disability due to shortness of breath (4.09vs2.04), constipation (3.97vs1.74), and difficulty concentrating (1.98vs1.25). Decedents also had a significant higher score (P<0.01) on self-reported loss of appetite (2.24vs1.91) and worse global quality of life rating (3.04vs2.64), compared to survivors. The patterns were similar in proxy-reported group and in the group with a combination of self-and proxy-reported interviews. Even in very late old age, disability and symptom burden increase with the approach of death.

